# Supplementary material for: Association of night-waking and inattention/hyperactivity symptoms trajectories in preschool-aged children
Source: Sci Rep. 2018 Oct 18;8:15412. doi: 10.1038/s41598-018-33811-2 (PMC6193981; doi:10.1038/s41598-018-33811-2)
Supplement: Supplementary file 1 — Supplementary data [file 41598_2018_33811_MOESM1_ESM.docx]

**Association of night-waking and inattention/hyperactivity symptoms trajectories in preschool-aged children**

**Supplementary data**

**Authors:**

Eve Reynaud PhD,^1,2,3^ Anne Forhan,^1,2^ Barbara Heude PhD,^1,2^ Marie-Aline Charles MD,^1,2^ and Sabine Plancoulaine MD, PhD,^1,2^

Distribution of covariates according to joint trajectories (N=1342), reporting % (N) or mean (SD)

|  | Rare night-waking (N=1073) | | | Common night-waking (N=269) | | |  |
| --- | --- | --- | --- | --- | --- | --- | --- |
| %(N) / mean(SD) | Low I/H^a^  N=571 | Medium I/H  N=384 | High I/H  N=118 | Low I/H N=59 | Medium I/H  N=154 | High I/H  N=56 | P^b^ |
| Parental characteristics |  |  |  |  |  |  |  |
| Household income |  |  |  |  |  |  | <0.001 |
| <1500 €/month | 7.2% (41) | 12.2% (47) | 16.1% (19) | 6.8% (4) | 16.9% (26) | 26.8% (15) |  |
| 1501-3000 €/month | 53.2% (304) | 61.5% (236) | 65.3% (77) | 72.9% (43) | 56.5% (87) | 62.5% (35) |  |
| >3000 €/month | 39.6% (226) | 26.3% (101) | 18.6% (22) | 20.3% (12) | 26.6% (41) | 10.7% (6) |  |
| Education (years)^c^ | 15.0 (2.3) | 14.2 (2.5) | 13.5 (2.3) | 15.1 (2.3) | 14.0 (2.5) | 13.4 (2.2) | <0.001 |
| History of childhood behavioral problem (yes) | 10.9% (62) | 16.7% (64) | 22.9% (27) | 11.9% (7) | 17.5% (27) | 25% (14) | <0.001 |
| Maternal depression (CES-D ≥23)^d^ | 6.0% (34) | 7.6% (29) | 9.3% (11) | 5.1% (3) | 6.5% (10) | 7.1% (4) | 0.24 |
| Maternal age at delivery (year) | 30.6 (4.6) | 29.7 (4.9) | 29.1 (4.4) | 30.4 (4.3) | 30.0 (4.9) | 27.7 (4.5) | <0.001 |
| Smoking during pregnancy (yes) | 15.4% (88) | 23.4% (90) | 29.7% (35) | 17.0% (10) | 27.9% (43) | 25.0% (14) | <0.001 |
| Child characteristics |  |  |  |  |  |  |  |
| Perinatal factors |  |  |  |  |  |  |  |
| Child gender (girl) | 53.4% (305) | 45.1% (173) | 28% (33) | 57.6% (34) | 45.5% (70) | 39.3% (22) | <0.001 |
| First child (yes) | 44.1% (252) | 48.7% (187) | 50% (59) | 44.1% (26) | 42.9% (66) | 66.1% (37) | 0.043 |
| Term at birth (weeks) | 39.3 (1.6) | 39.2 (1.8) | 39.4 (1.7) | 39.4 (1.3) | 39.1 (2) | 39.3 (1.7) | 0.34 |
| Lifestyle and sleep habits at age 2 |  |  |  |  |  |  |  |
| Collective care arrangement (yes) | 24.3% (139) | 22.4% (86) | 9.3% (11) | 28.8% (17) | 16.2% (25) | 12.5% (7) | <0.001 |
| Television viewing (h/day) | 0.6 (0.6) | 0.8 (0.7) | 0.7 (0.8) | 0.8 (0.6) | 0.8 (0.7) | 0.9 (0.9) | <0.001 |
| [Inadvisable](http://www.linguee.fr/anglais-francais/traduction/inadvisable.html) sleep habits | 8.4% (48) | 10.9% (42) | 7.6% (9) | 6.8% (4) | 13% (20) | 5.4% (3) | 0.48 |
| Night-sleep duration (h/day) | 11.2 (0.8) | 11.1 (0.8) | 11.1 (0.7) | 10.9 (0.7) | 11.0 (0.8) | 10.9 (1.0) | 0.001 |

^a^ I/H inattention/hyperactivity

^b^ Global type 3 p-value obtained from unadjusted logistic regression, with the joint “rare night-waking and low inattention/hyperactivity trajectories” as reference

^c^ Number of years of schooling starting from first year of primary school e.g. 12 years corresponds to having completed high school

^d^ Center of Epidemiologic Studies Depression-Scale, cutoff validated in a French population, measured during pregnancy
